# An epidemiological study of food allergy in a regional hospital in Ireland

**DOI:** 10.1186/2045-7022-5-S3-P92

**Published:** 2015-03-30

**Authors:** Jennifer Cox, John Fitzsimons, A Nadeem Khan

**Affiliations:** 1 Department of Paediatrics, Our Lady of Lourdes Hospital, Drogheda, Ireland

## Introduction

Food allergy is rapidly becoming a significant burden on the paediatric population of developed countries.[1,2] Type 1 allergy causes much anxiety and leads to some of the very acute and distressing presentations to paediatric A&E. Misconception and fears regarding the use of adrenaline preparations is one of the causative factors, although there is a huge evidence base that shows the safety and effectiveness of these preparations in managing the true and suspected cases of type 1 allergy.[3]

In the Paediatric unit in Our Lady of Lourdes Hospital, Drogheda there is an allergy clinic which runs as an out-patient service.

Our aim was to establish a database of children who presented to these clinics and record the allergens (both food and aeroallergens) to which they had a positive reaction. We included those that had a positive skin prick test or radioallergosorbent test (RAST), or both. It is only by creating such a database that we can determine both the scale of the problem in our area and the adequacy of services provided. Using this information we could proceed further to audit our services and make any improvements in the future.

The aim of the allergy register was to:

• Obtain information about 1) the number of children presenting to our allergy clinic and 2) the prevalence of food allergy within this group and the type of allergens (nut, fish, milk, egg).

• Determine the number of children with associated atopic conditions (asthma, eczema, hayfever).

• Record the nature of reaction to the various allergens.

• Record the patients who had been given adrenaline pens so that timely replacements can be provided in the future.

## Methods

Information was collected retrospectively from the clinic letters of the allergy clinic from January 2011 to December 2013. Information collected included personal biodata of the patients (age, sex etc), their confirmed allergies (either through skin prick testing or RAST), the number of those prescribed adrenaline injection or anti-histamine tablets and associated atopic conditions (eczema, asthma, hayfever).

## Results

There were a total of 490 children who attended the allergy clinic in the three year period. Of these, 62% were males, 38% were females. Approximately half were below five years of age (56%). Of the patients that presented to our clinics, 64% had confirmed food allergies through skin prick testing or blood testing (RAST) or both. True symptoms of anaphylaxis were reported in only 10% of attendees, while other documented reactions included skin rashes, urticaria and gastrointestinal symptoms. Food allergy to egg, milk and nuts were the most common presentations, comprising more than 80% of the cases. Adrenaline pen prescriptions were provided to 35% of patients. A significant number of children had associate atopic conditions; asthma was reported in 41.17%, with hay fever in 28.57% and eczema in 53.78%.

## Conclusion

The rising prevalence of food allergies is reflected in the increasing number of cases presenting to the paediatric allergy clinic in our hospital. More than 80% of the patients were allergic to egg, milk, peanut and tree nuts. Approximately 30% of these patients had been prescribed an adrenaline pen for use in case of anaphylaxis.

A number of recommendations could be implemented from the information collected from this audit. For example, the data collected in this allergy register could be used to form a standardised database and would be useful for follow up clinics. Patients with adrenaline prescriptions could be flagged and contacted to ensure that they had updated prescriptions available and are confident in their technique of administering the adrenaline.

## References

[B1] Scott Sicherer H Epidemiology of food allergy Journal of Allergy and Clinical Immunology 2011 127 3 594 602 10.1016/j.jaci.2010.11.044 21236480

[B2] Rona,Roberto J The prevalence of food allergy: A meta-analysis Journal of Allergy and Clinical Immunology 2007 120 3 638 646 10.1016/j.jaci.2007.05.026 17628647

[B3] Lieberman P Nicklas RA Oppenheimer J The diagnosis and managemet of anaphylaxis practice parameter: 2010 update Journal of Allergy and Clinical Immunology 2010 126 477 480 10.1016/j.jaci.2010.06.022 20692689

